# To identify Harlequin syndrome in patients with venoarterial extracorporeal membrane oxygenation using radial near-infrared spectroscopy

**DOI:** 10.1186/s13054-023-04793-z

**Published:** 2024-01-08

**Authors:** Yongwei Yu, Xing Fang, Zhipeng Xu, Tong Li, Jueyue Yan

**Affiliations:** Department of Critical Care Medicine, The First Affiliated Hospital, Zhejiang University School of Medicine, No. 79 Qingchun Road, Hangzhou, 310003 Zhejiang Province China

**Dear Editor,**

Harlequin syndrome is a serious complication in peripheral VAECMO that occurs when left ventricular function begins to recover while oxygenation of the lungs is poor [1]. The incidence has not been clearly stated, but it has been reported to be 8.8% [2]. When Harlequin syndrome occurs, the heart and brain are directly damaged. Therefore, early identification and detection of Harlequin syndrome is the focus of the whole treatment.

The right radial artery is a branch of the brachiocephalic trunk, so the blood oxygen content of the radial artery is often monitored to monitor the occurrence of Harlequin syndrome. At present, two methods are used to detect blood oxygen saturation in clinical practice. The first is invasive blood gas analysis, which uses biochemical methods to obtain its oxygen saturation. Blood gas analysis in detection of blood oxygen saturation is the only "gold standard." The second is to measure pulse oxygen saturation (SpO2), a noninvasive optical method that has been widely used in clinical practice. The sensor is clamped at the fingertip, and the detection principle is based on the pulse of the fingertip artery, that is, the pulse component of the emitted light attenuated by the fingertip is extracted and calculated. However, arterial blood gas analysis is an invasive operation with high cost, and the information obtained is lagging, which cannot be continuously monitored. The detection principle of SpO2 depends on the pulse of the fingertip artery, so it is not suitable for hypoperfusion and ECMO advection. Near-infrared spectroscopy (NIRS) is a noninvasive, real-time and continuous method for the measurement of blood oxygen parameters in human tissues, independent of arterial pulse. NIRS is commonly used to monitor brain tissue oxygenation. However, some studies have suggested that there is a weak correlation between brain tissue oxygenation and physiological variables of the whole brain tissue. Only when the physiological variables of the whole brain change significantly, brain tissue oxygenation will change correspondingly [3], and irreversible damage may have occurred in the brain tissue. Anatomically, the radial artery and the right internal carotid artery originate from the brachiocephalic trunk, and the oxygenation of the right radial artery can reflect the oxygenation of the right internal carotid artery. According to the principle of NIRS, we think it also suitable for radial artery blood oxygenation monitoring area. After repeated comparisons of different parts, it was finally found that rSO2 at the radial artery pulse was the most sensitive to the change of oxygen partial pressure (Fig. 1a). After excluding patients with localized arterial disease, we included a cohort of patients whose hemoglobin was maintained at 9–10g/dl. Our team find the strong correlation between radial artery blood gas oxygen saturation and rSO2 (Fig. 1b).

**Fig. 1:**
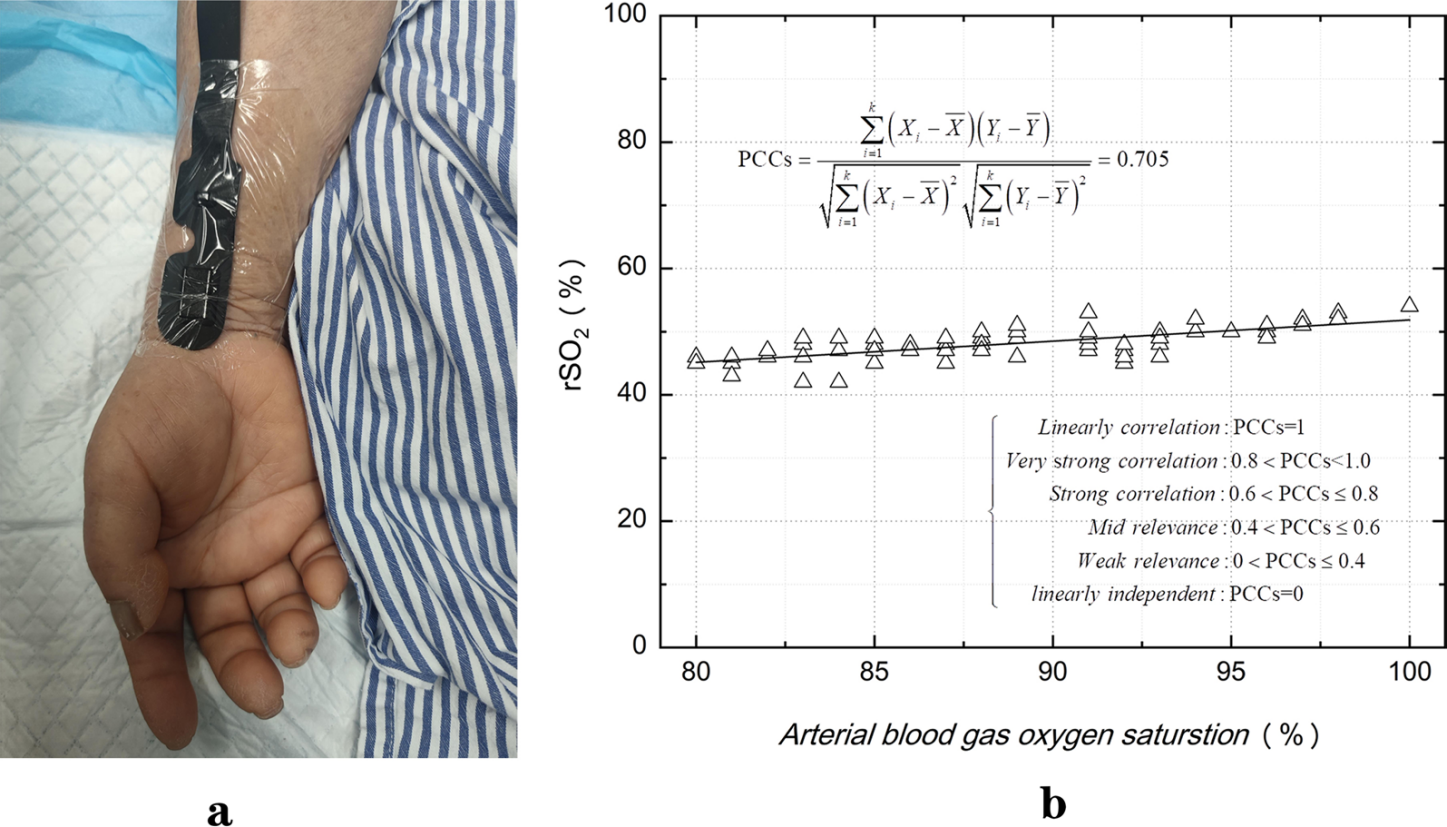
**a** In the radial pulse way of tissue oxygen monitoring. **b** Correlation between radial artery blood gas oxygen saturation and rSO2

We must acknowledge that rSO2 cannot be equated with actual arterial oxygen saturation. It actually reflects the balance between oxygen supply and demand in local tissues. Although the value of rSO2 may vary under different circumstances, its changing trend is clear. Since the patient with VAECMO advection is routinely sedated and analgesic, it can be assumed that his local metabolism will not change in the short term. In the case of stable hematocrit, there is a close correlation between rSO2 and oxygen saturation. We suggest that arterial blood gas analysis be performed for calibration and validation when rSO2 is initiated and its trend changes substantially. We believe that using NIRS for VAECMO patients the right side of the radial artery blood supply region monitoring is beneficial to early detection of the Harlequin syndrome.

## Data Availability

The datasets used and/or analyzed during the study are available from the corresponding author on reasonable request.
